# Stumbling across the Same Phage: Comparative Genomics of Widespread Temperate Phages Infecting the Fish Pathogen *Vibrio anguillarum*

**DOI:** 10.3390/v9050122

**Published:** 2017-05-20

**Authors:** Panos G. Kalatzis, Nanna Rørbo, Daniel Castillo, Jesper Juel Mauritzen, Jóhanna Jørgensen, Constantina Kokkari, Faxing Zhang, Pantelis Katharios, Mathias Middelboe

**Affiliations:** 1 Marine Biological Section, University of Copenhagen, DK-3000 Helsingør, Denmark; panos.kalatzis@bio.ku.dk (P.G.K.); nanna.rorbo@bio.ku.dk (N.R.); danielcastillobq@gmail.com (D.C.); kcq442@alumni.ku.dk (J.J.M.) ; johannajorgensen88@gmail.com (J.J.);; 2 Institute of Marine Biology, Biotechnology and Aquaculture, Hellenic Centre for Marine Research, Crete 71500, Greece; dkok@hcmr.gr (C.K.); katharios@hcmr.gr (P.K.); 3 Beijing Genomics Institute (BGI) Park, No.21 Hongan 3rd Street, Building NO. 7, Yantian District, Shenzhen 518083, China; jason.zhang@genomics.cn

**Keywords:** bacteriophages, temperate, *Vibrio anguillarum*, Siphovirus, genetic similarity, omnipresent, lysogenic conversion.

## Abstract

Nineteen *Vibrio anguillarum*-specific temperate bacteriophages isolated across Europe and Chile from aquaculture and environmental sites were genome sequenced and analyzed for host range, morphology and life cycle characteristics. The phages were classified as Siphoviridae with genome sizes between 46,006 and 54,201 bp. All 19 phages showed high genetic similarity, and 13 phages were genetically identical. Apart from sporadically distributed single nucleotide polymorphisms (SNPs), genetic diversifications were located in three variable regions (VR1, VR2 and VR3) in six of the phage genomes. Identification of specific genes, such as N6-adenine methyltransferase and lambda like repressor, as well as the presence of a tRNA^Arg^, suggested a both mutualistic and parasitic interaction between phages and hosts. During short term phage exposure experiments, 28% of a *V. anguillarum* host population was lysogenized by the temperate phages and a genomic analysis of a collection of 31 virulent *V. anguillarum* showed that the isolated phages were present as prophages in >50% of the strains covering large geographical distances. Further, phage sequences were widely distributed among CRISPR-Cas arrays of publicly available sequenced *Vibrio*s. The observed distribution of these specific temperate Vibriophages across large geographical scales may be explained by efficient dispersal of phages and bacteria in the marine environment combined with a mutualistic interaction between temperate phages and their hosts which selects for co-existence rather than arms race dynamics.

## 1. Introduction

*Vibrios* are a genetically and metabolically diverse group of marine heterotrophic bacteria that plays significant roles in marine biogeochemical cycling [1]. The genus is globally distributed in marine and brackish environments and occurs as both free-living and surface-associated communities ranging from coastal areas to the open sea [1,2,3]. The group contains several pathogens, including *Vibrio anguillarum*, which infects more than 50 species of fish, mollusks and crustaceans, causing vibriosis [4], a devastating disease affecting the aquaculture industry worldwide. *V. anguillarum* consists of both pathogenic and non-pathogenic strains [5,6], and the mechanism of pathogenesis of *V. anguillarum* is not completely understood yet. Recent advancements in genome sequencing have started to shed light in putative bacterial virulence and fitness factors including exotoxins, adherence/colonization proteins, invasion, capsule and cell surface components as well as iron uptake system [7,8].

Virulence in *Vibrios* can also be associated with the expression of prophage-encoded genes through the process of lysogenic conversion [9,10,11]. A well-known example of prophage generated virulence is the human pathogen *V. cholerae*, in which the key toxins (CTX and Zot) are encoded by specific prophages [12,13]. More recently, these prophage-associated toxins have also been identified in environmental *Vibrios* (e.g., *V. coralliilyticus*), indicating that prophage-encoded genes are disseminated between environmental *Vibrio* populations [14]. In addition, in the fish pathogen *V. harveyi,* the prophage VHML conferred virulence by lysogenic conversion [15,16] and recent bioinformatic analyses have identified numerous phage genes potentially encoding virulence and fitness factors in environmental *Vibrio* strains [14,17]. Therefore, there is increasing evidence that lysogenic conversion plays a significant role in shaping environmental *Vibrio* populations contributing to their functional and genetic diversity. In addition to lysogenic conversion, prophages can also affect host properties by serving as anchor points for genomic rearrangements [18], by disrupting critical genes or operons during integration and excision [19] and by preventing infection by similar phages with superinfection exclusion mechanisms [20]. Further, phage-derived bacterial immunity can be obtained by phage-encoded interactions with restriction-modification (RM) system, such as expression of methyltransferases [21,22]. 

The presence of prophages in marine bacteria has also been connected with more subtle benefits for the bacterial host. Marine prophages can suppress unneeded metabolic activities of their host under unfavorable environmental conditions, serving as energy saving and survival enhancing tool [23]. Thus, *Vibrio* prophages and temperate phages constitute potentially a major reservoir of functional traits in the marine environment, including virulence. The mobilization of these phage-encoded properties may be a key driving force for dissemination of such properties in environmental marine *Vibrio* communities.

Bacteriophages can display biogeographical patterns following their hosts’ geographical patterns [24] and both cosmopolitan phages with a worldwide distribution [25], and phages constrained to a specific environment have been observed [9,26]. Previous studies have found indications that populations of genetically related vibriophages are widely distributed over large geographic distances in the oceans [27]. However, little is known about the geographical distribution of specific vibriophages. The diversity of bacteriophages can be high when examined locally but when a more global approach is attempted, diversity can be relatively limited due to the phages ability move among different biomes [27,28,29]. 

Studies on *V. anguillarum*-specific bacteriophages have so far mainly focused on lytic phages and their potential use in phage therapy application [30,31,32] while very little is known about the role of temperate phage-encoded virulence and other fitness factors as drivers of functional dynamics in *V. anguillarum*. Improving our understanding of how phages may influence the genomic and phenotypic characteristics of *V. anguillarum* thus requires more detailed knowledge of the genetic properties and dispersal of temperate phages infecting this pathogen as well as their distribution as prophages in the host community.

In this study, we report the complete nucleotide sequences, annotations and genome comparison of 19 *V. anguillarum*-specific temperate bacteriophages isolated from Denmark, Norway, Greece and Chile using both aquaculture and environmental sites. Further, we analyze the distribution of the temperate phages as prophages in a collection of 31 virulent *V. anguillarum* strains and test their ability to lysogenize *V. anguillarum*. The bacteriophage sequences were mapped against the available CRISPR-Cas arrays of the *Vibrio* genus in order to assess past events of phage-bacterial interactions leading to spacer acquisition. The geographical distribution and genomic characterization, along with the phage morphology, host range and kinetic parameters of selected phages unraveled a novel insight into a group of temperate phages, designated H20-like phages and their potential for lysogenic conversion and interactions with *V. anguillarum* across large spatial scales.

## 2. Materials and Methods

### 2.1. Bacterial Strains and Growth Conditions

The *V. anguillarum* strains A023, T265, BA35 and VaKef were used as hosts for bacteriophage isolation. The host strains are part of a *V. anguillarum* collection, which consists of 31 well characterized and whole genome sequenced strains [33]. All bacterial strains were stored at −80 °C in Luria-Bertani (LB) medium (Invitrogen, Carlsbad, CA, USA) supplied with 15% glycerol. For proliferation, the strains were inoculated in LB medium and incubated at 25 °C with constant agitation.

### 2.2. Isolation, Purification and Propagation of Bacteriophages

More than 100 aquaculture and environmental marine water samples have been collected from Denmark, Norway, Greece and Chile and isolation of phages was performed using standard enrichment methodology [34] with minor modifications. The bacterial strains used for phage isolation were added separately to the water samples, supplemented with 10% LB medium. Samples were incubated overnight at 25 °C with constant agitation, following centrifugation at 6000× *g* for 10 min. Supernatants were filtered through 0.22 μm syringe filters (Whatman, Maidstone, UK) and the presence of phages was examined with plaque assay using the double-layer agar method [35]. Briefly, 100 µL filtrate was added to 300 μL liquid cultures of each of the bacterial strain that were used for phage isolation. The mixture was then added to 4 mL melted soft agar (0.4% agar and 1% sea salts (Sigma-Aldrich, Saint Louis, MO, USA)) and poured onto LB 1.5% agar plates. Following overnight incubation at 25 °C, the plates were inspected for plaque forming units (pfu) on the host bacterial lawn. Single phage plaques were picked and purified by 5× re-plating. In this way, 19 clonal phage isolates were obtained from individual water samples (Table 1). Proliferation of bacteriophage isolates was conducted in 50 mL LB medium, by mixing the bacterial host strain at its early exponential phase with the corresponding bacteriophages at a multiplicity of infection (MOI) of 10, and incubating the mixture overnight at 25 °C with constant agitation. Following centrifugation and filtration through 0.22 μm syringe filters, the 19 final bacteriophage stocks were titered and stored at 4 °C.

Water samplings did not require any special permission. 

### 2.3. Morphology, Life Cycle and Host Range of Bacteriophages

Virion morphology of two of the H20-like bacteriophages, φVaK and pVa-7, was determined under Transmission Electron Microscopy (TEM) observation at the facilities of University of Crete, Greece. Samples were prepared on collodium copper grids, negatively contrasted with 2% uranyl acetate, and examined by JEOL JEM2100 electron microscope at 80 kV, applying instrumental magnification of 120,000. The morphology of the phages (H20 and H8) was determined previously [36].

One-step growth experiments were performed with φH20, pVa-3, φCLA and φP3 by adding the phages to 1 mL of their bacterial host *V. anguillarum* strain BA35 (MOI:0.001) at early exponential phase in LB medium and incubating at 25 °C for 15 min. Following centrifugation at 6000× *g* for 10 min, supernatants containing free, unattached phages were discarded, while phages which had attached to the bacterial hosts were pelleted. The bacterial cells were then resuspended in 20 mL medium containing 1% Tryptone (Difco, Livonia, MI, USA), 0.5% Yeast extract (Difco), and 2% sea salts (Sigma-Aldrich). Setting that time point as t = 0, 20 μL drops of serial dilutions were spotted on bacterial lawns prepared on LB agar plates every 10 min for total duration of 130 min. Plaque forming units in the spotted areas were counted after overnight incubation at 25 °C. One-step curves were plotted from 3 individual experiments performed for each of the 4 bacteriophages assayed. 

Phage adsorption was evaluated for φH20, pVa-3, φCla and φP3, using 6-well plates each containing 3 mL of *V. anguillarum* strain BA35 liquid culture at early exponential phase of growth in LB medium. Each bacteriophage tested was added to 3 wells (triplicates) applying a MOI of 0.001. Samples of 200 μL were collected every 5 min, followed by centrifugation at 10,000× *g* for 2 min. Drops of 20 μL containing un-adsorbed phages were taken from the supernatant and spotted on the host bacterial lawn as above. The total duration of the experiment was 30 min and plaque forming units were counted after overnight incubation at 25 °C. The phage adsorption constant was calculated from the decrease in the free, unattached phages over the incubation period [37,38].

The lytic spectrum (host range) of the isolated bacteriophages was assessed by spotting them on the bacterial lawns of the 31 *V. anguillarum* pathogenic strains collection. Twenty-microliter drops of each phage concentrate were spotted in triplicate on bacterial lawns followed by overnight incubation at 25 °C. 

### 2.4. DNA Extraction and Sequencing 

DNA was extracted from all 19 isolated bacteriophages using high titer samples (10^10^ pfu·mL^−1^). The viral particles were concentrated by adding poly-ethylene glycol to the 4 °C phage stocks, followed by centrifugation (20,000× *g*, 1 h) and resuspension of the phage pellet in 100 μL of NaCl solution (150 mM). QIAamp DNA Blood Mini Kit (QIAGEN, Hilden, Germany) was used for the bacteriophage genome extraction, according to the manufacturer’s protocol. The concentration of phage DNA was quantified by NanoDrop (Thermo Fisher Scientific, Waltham, MA, USA) and the DNA quality was assessed in an agarose gel.

Genome sequencing for all isolated bacteriophages was conducted using Illumina Hi Seq 2000 sequencer (Illumina, San Diego, CA, USA) at Beijing Genomic Institute (Shenzhen, Guangdong, China) using pair-end read sizes of 100 bp. Sequencing process, library construction and trimming of contaminated reads and primers, were performed in accordance with the manufacturer’s protocols. De novo assembly of the produced reads was done by Geneious algorithm, with Geneious software bioinformatics platform R9 version [39], resulting in single contigs for all phages. Short and low-coverage contigs were discarded.

### 2.5. Annotation, Comparative Genomics, and Phylogeny

The genes of the sequenced bacteriophages were predicted by Glimmer 3 [40] and the open reading frames (ORFs) were generated. Annotations of the genes were performed automatically by Rapid Annotation Subsystem Technology (R.A.S.T.) [41,42]. Both annotated ORFs and hypothetical proteins ORFs that represented coding sequences (CDSs), were crosschecked manually by protein Basic Local Alignment Tool (BLASTP) and by Protein Fold Recognition Server, Phyre^2^ [43,44], defining the gene functions both by genetic similarity and by protein structure. The presence of tRNA was assessed by the online tool ARAGORN [45]. Alignment of whole viral genomes was conducted by progressive MAUVE algorithm [46] using the Geneious software bioinformatics platform [39]. The direct terminal repeats (DTR) that were detected at the right and left ends of each phage contig marked the physical ends of the phage genomes (151 bp for φVaK and 143 bp for the rest phages). Additionally, attL and attR were characterized by the sequence TCGTGATTCCTTGC(T)CACCG CCACATCCAAGCCTCTTG(A)GGTATCAAGAGGCTTTATTTTATCTGACAGACCCCGCAAT(T) and direction of all sequences is attL → attR. Phylogenetic analyses of both the isolated phages and the *V. anguillarum* prophages included in the study, were conducted by Geneious Tree Builder, using Neighbor-Joining method and Tamura-Nei genetic distance model (1000 bootstraps).

### 2.6. CRISPR Arrays and Prophage Detection in Vibrio

All publicly available *Vibrio* genomes and downloaded from the National Center for Biotechnology Information (NCBI) GenBank [47]. The presence of CRISPR arrays in the *Vibrio* genomes was evaluated in 1185 genomes representing 64 different *Vibrio* species, using CRISPR Finder online tool [48]. Further, potentially inducible intact prophages were detected in the genomes of our *V. anguillarum* collection [34] using the online prophage finder tool, PHAST [49]. 

### 2.7. Integration of Phages in *V. anguillarum* Strain BA35

The ability of the isolated temperate phages to integrate into the bacterial host’s genome was evaluated by coculturing *V. anguillarum* strain BA35 with φH20. Triplicate bottles with 48 mL LB medium were inoculated with the addition of host strain BA35 and φH20 (MOI: 50), incubated at 25 °C for 12 h with agitation. Bacterial growth was monitored by measuring optical density at 600 nm (OD_600_) every hour using Novaspec Plus Visible Spectrophotometer (Amersham Biosciences, Uppsala, Sweden). Exponential growth was observed after 10–12 h of incubation and triplicates of 1 mL were sampled and serially diluted in SM buffer (Sodium Magnesium; 100 mM NaCl, 8 mM MgSO_4_·7H_2_O, 50 mM Tris-Cl, 0.01% gelatin, pH: 7.5). Dilutions were poured onto LB agar plates and the following day, 50 bacterial colonies were isolated and recultured at 25 °C. The ability of the bacteriophage φH20 to lysogenize its host was assessed by Polymerase Chain Reaction (PCR) with 3 sets of specific primers (Supplementary Table S1). They were able to target and propagate 3 specific phage genes: a structural tail protein gene, a terminase large subunit gene and a hypothetical protein gene. All 50 isolated bacterial strains were washed 5 times with SM buffer in order to eliminate the possibility of a false positive reaction caused by any remnant phage particles. A triple positive PCR would confirm the lysogenization. 

### 2.8. GenBank Accession Numbers

The GenBank accession numbers for the new sequenced bacteriophages are: KX581090, KX581091, KX581092, KX581093, KX581094, KX581095, KX581096, KX581097, KX581098, KX581099, KX581100, KY658673, KY658674, KY658675, KY658676, KY658677, KY658678, KY658679, and KY658680. Since all 19 phages were genetically similar, they were designated as H20-like phages named after φH20, which was isolated and described as the first [36].

## 3. Results

### 3.1. Isolation and Characterization of Bacteriophages

Sixteen bacteriophages were isolated in the current study using two *V. anguillarum* strains as hosts: strain BA35 (φΡ2, φP3, pVa-1, pVa-2, pVa-3, pVa-4, pVa-5, pVa-6, pVa-7, pVa-8, φCLA, φHer, φLen, φPel and φStrym) and strain VaKef (φVaK). Further, three phages were isolated in a previous study [36] using three different hosts: BA35 (φΗ20), T265 (φΗ8) and A023 (φΗ2). None of the used host strains contained H20-like prophages, implying that the isolated phages originated from the water samples. All phages produce similar 1 mm plaques on their host bacterial lawns. They were isolated from four different countries (Denmark, Norway, Greece and Chile), both from aquaculture and environmental marine water samples. Their genome sizes ranged between 46,006 and 54,201 base pair (bp) with a similar GC content of 43%–43.1% and with a number of ORFs between 76 and 94 (Table 1). 

**Figure 1:**
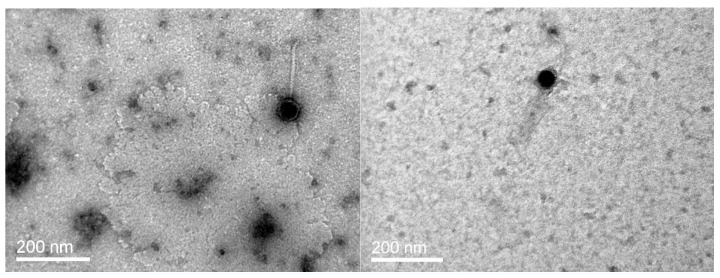
Transmission electron microscopy micrographs of bacteriophages: φVaK (**left**); and pVa-7 (**right**) classifying them to Siphoviridae family.

In addition to the genomic characterization, a selection of the H20-like phages group was further analyzed for virion morphology and life cycle parameters. The observed morphology of the virions under TEM classified the bacteriophages to the Siphoviridae family. Virions had a long, non-contractile tail of about 150 nm and a head of approximately 50 nm in diameter (Figure 1). 

One-step growth and phage adsorption curves have been generated to determine the life cycle parameters of the bacteriophages (Supplementary Figure S1A,B). Latency time, burst size and adsorption constant ranged from 50 to 70 min, 100 to 145 virions per cell and 8.6 × 10^−8^ to 1.2 × 10^−7^, respectively (Table 2).

### 3.2. Host Range Analysis and Phylogeny

**Figure 2:**
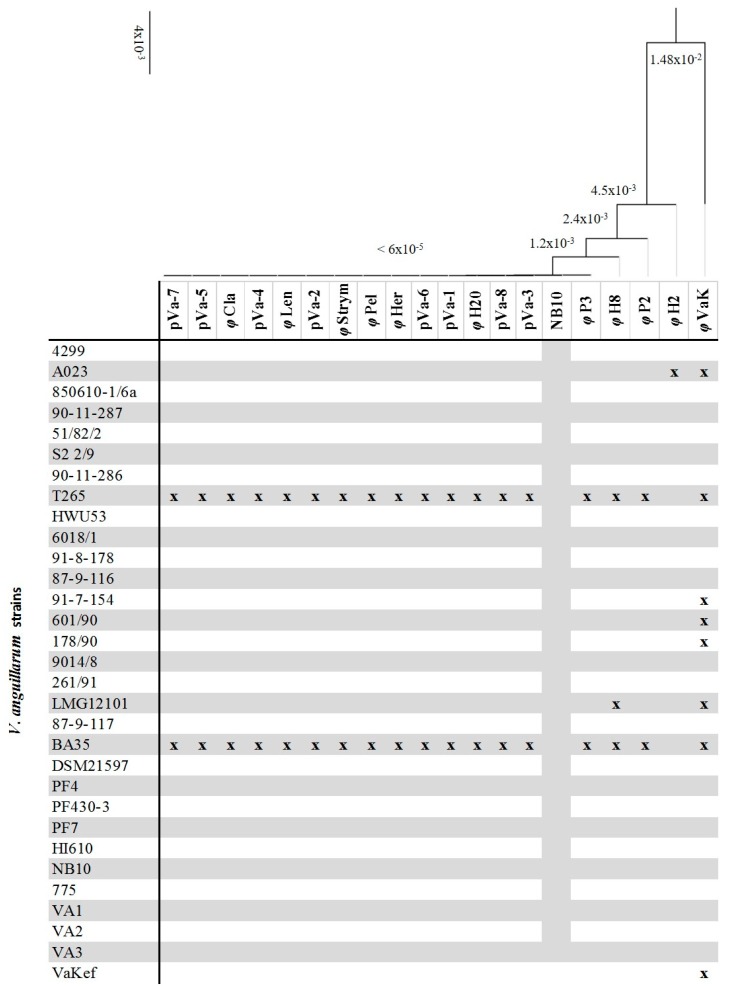
Host range of the 19 bacteriophage isolates against 31 pathogenic *V. anguillarum* strains. Blocks marked with x, indicate the infection of the bacteria by the corresponding bacteriophage isolate. The phylogenetic tree based on the whole genomes of the 19 sequenced phages of the current study (Neighbor-Joining method). The prophage from *V. anguillarum* strain NB10 was included as a reference phage. The branch labels indicate the substitutions per site.

Thirty-one pathogenic strains of *V. anguillarum* were used to determine the lytic spectrum of the 19 bacteriophage isolates (Figure 2). Seventeen out of 19 phages had almost identical host range with only one minor difference in the case of φΗ8. Two phages showed a different host range: φH2 only infected strain A023, whereas bacteriophage φVaK had a broader host range and infected eight out of 31 bacterial strains tested. According to the phylogenetic analysis of the 19 isolated bacteriophages, φP3, φH8, φP2, φH2 and φVaK had a higher number of substitutions per site. The combined host range table and the phylogenetic tree of the bacteriophages (Figure 2) indicated that the differences in the lytic spectrum are accompanied by differences at the genetic level. Especially in the case of φVaK, where most genetic differences are observed, the lytic spectrum is much broader than the others.

### 3.3. Comparative and Functional Phage Genomics

**Figure 3:**
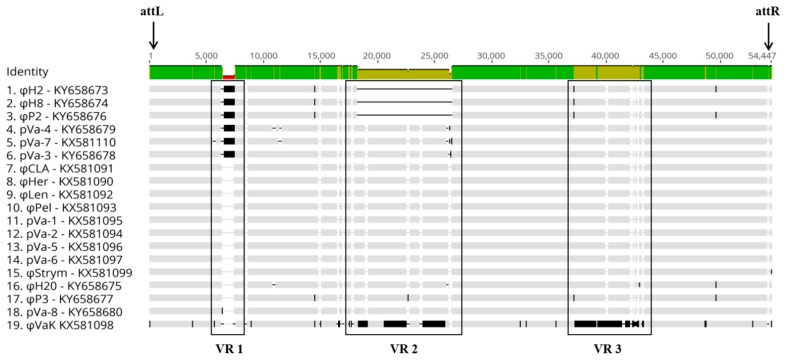
Genomic alignment of the 19 newly sequenced temperate bacteriophages. The black boxes indicate the variable regions (VR 1, VR 2 and VR 3). Single nucleotide polymorphisms (SNPs) and genetic differences are found sporadically in the genomes as black vertical lines either thin or thick depending on the length of the differences. Genome gaps are depicted with the black horizontal lines. The physical ends of the genomes are indicated by direct terminal repeats (DTRs) following the direction of attL → attR.

The genomes of all 19 bacteriophages closely resemble a prophage genome which is present in the *Vibrio anguillarum* strain NB10 (GenBank No. LK021130) [50]. Additionally, H20-like bacteriophages were also found to be a part of several *V. anguillarum* genomes that have been recently released in the NCBI GenBank [34]. However, genomic comparisons of the phages have revealed some differences at the genetic level, which are depicted in an aligned sequence graph (Figure 3). 

SNPs are sporadically distributed through the 19 phage genomes. However, there are three variable regions, named VR1, VR2 and VR3, which show systematic modifications among phages (Figure 3). In phages φP2, φH8 and φH2, the biggest part of VR2 is completely missing, whereas VR1 is only present in phages φP2, φH8, φH2, pVa-3, pVa-4 and pVa-7. As a result of these genomic modifications, the H20-like bacteriophages demonstrate a range of genome sizes, between 46-kb and 54-kb (Table 1). 

Apart from the presence/absence of VR1 and VR2, most of the phages are genetically homogenous with the exception φVaK, where both VR1 and VR3 are very diverse compared to all other phage genomes, while VR1 is missing. SNPs in the genome of φVaK are also more frequent compared to the other bacteriophages. These genomic differences seem to be reflected in the phage’s phenotype, since the host range of this phage is much broader than the others (Figure 2). 

**Figure 4:**
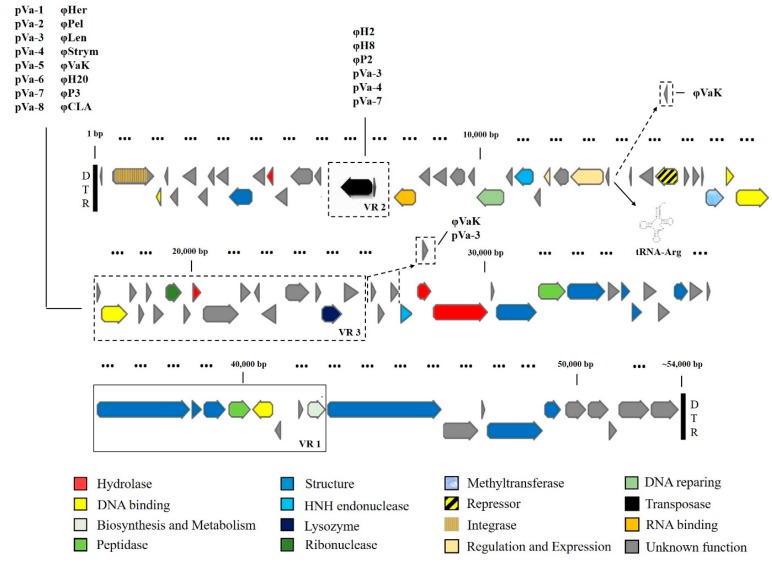
The core- and accessory- gene content of the H20-like bacteriophages. Core-gene content is composed by 74 genes shared by all 19 bacteriophages. Accessory-gene content is indicated by dashed lines, with VR1 and VR2 representing the main components. The bacteriophages that contain the accessory gene blocks VR1 and/or VR2 are correspondingly indicated in columns beside. Two more hypothetical proteins complement the accessory gene content and their corresponding carrier phages (pVa-3 and φVaK) are also indicated. DTRs mark the physical ends of the genome (bold vertical lines).

For most of the genes in the H20-like phages, the function remains unknown. However, taking into consideration both the genetic similarity and the protein folding structure of the viral gene products, it was possible to define the predicted function of approximately one third of the viral genes. The viral genes were predicted and then annotated based on their genetic similarity with the NCBI GenBank database, mainly referring to the prophage of strain NB10 annotation. Additional information on gene function that came from their protein folding pattern, determined by amino acid composition, contributed significantly in complementing the annotation of the bacteriophages. Thus, 15 gene functions and protein families were attributed to the one third of the viral genes, with structural and DNA binding proteins being mostly represented. Seventy-four genes compose the core-gene content of all 19 bacteriophages and this is the common basis on which accessory genes are added in some of the phage genomes (Figure 4). These 74 genes encode all the structural proteins, most of the DNA binding proteins and hydrolases and all the proteins related to regulation of gene expression, biosynthesis and metabolism, peptidase and lysozyme activity, ribonuclease activity, HNH endonucleases, RNA binding and DNA repairing. The presence of an integrase gene suggests that the H20-like bacteriophages are temperate phages able to be integrated in their host’s genome, as it has happened in the case of the *V. anguillarum* NB10 prophage. Repressor, integrase, methyltransferase and tRNA^Arg^, are also part of the core gene content present in all 19 bacteriophages. The core genome thus supports the functionality of the H20-like bacteriophages. However, 21 different accessory genes are present in some of the bacteriophage genomes. VR1 and VR2 are the main genomic parts of the accessory genome since they possess two and 17 genes, respectively. VR1 has a transposase and a putative 5-methycytocin specific restriction enzyme and VR2 has 13 genes of unknown function, two hydrolase, one DNA binding and one lysozyme activity proteins (Figure 4). The presence of accessory genes was not associated with any of the measured phenotypic properties.

In the case of φVaK, VR2 and VR3 are the most diverse compared to the other homogenous phage genomes. However, the vast majority of the changes are silent and do not translate into any differences in the predicted gene function at the amino acid level. 

The detailed annotations for the 19 temperate bacteriophages are listed in the Supplementary Table S2. 

### 3.4. *Vibrio* CRISPR Spacers in H20-Like Phages

Although H20-like bacteriophages were isolated from distant and different locations, their genetic differences are minor. In order to assess the interaction of H20-like bacteriophages with *Vibrio* hosts in general, a matrix containing the CRISPR arrays from all the published *Vibrio* genomes was composed. Keeping the cut-off value of >80%, 10 CRISPR spacers from three different *Vibrio* species were found to match with the consensus genome of the H20-like phages (Supplementary Table S3), implying a *Vibrio*–H20-like phage contact at some point through evolutionary time. Furthermore, the mapping of H20-like phage genomes against the two publicly available CRISPR arrays of *V. anguillarum* strain PF7, led to 24 spacers matching with >80% similarity. Eighteen of them were >95% similar, whereas eight of them were 100% identical to genomic parts of the H20-like phages. The spacers matched against several regions in the phage, and were randomly scattered in the genome. Additionally, in the case of *V. navarrensis* (ATCC 51183), the spacer 21 matches 100% with its corresponding part in the H20-like phage genome, whereas 9 more spacers mapped with >80% similarity. In order to assess the presence of H20-like prophages in *V. anguillarum,* the genomes of the bacterial collection of *V. anguillarum* strains were analyzed. The sequences of 17 H20-like prophages were bioinformatically detected as integrated in the 31 *V. anguillarum* genomes (Table 3). 

### 3.5. H20-Like Prophages Presence in *V. anguillarum*

**Figure 5:**
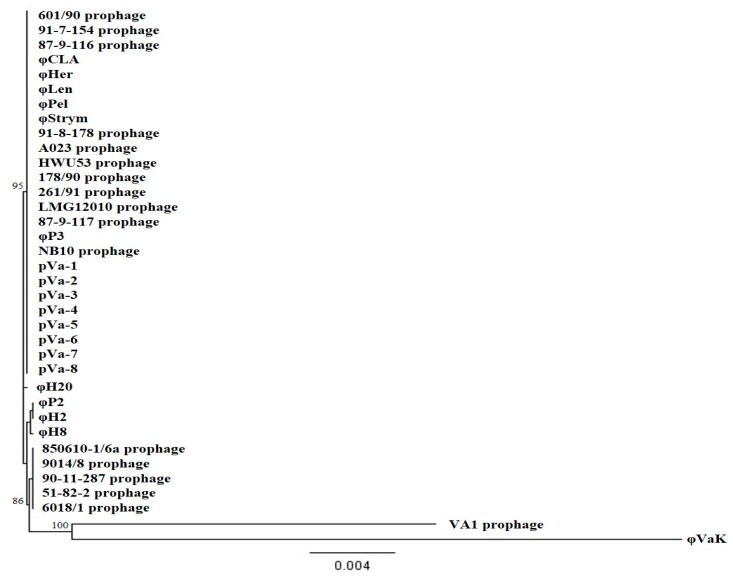
Phylogenetic tree (Neighbor-Joining method) based on the whole genomes of the 19 temperate bacteriophage isolates of the current study and 17 prophages which were bioinformatically detected in the genome of their *V. anguillarum* hosts. The branch labels indicate the percentage of statistical support.

According to the phylogenetic tree that was constructed from the identified prophages and isolated temperate phages, five out of 17 prophages and three out of 19 free-living phages are forming two different small monophyletic groups. However, 11 out of 17 prophages and 15 out of 19 free-living temperate phages are clustered together in a genetically and statistically robust and homogenous taxon, independent of their origin. Bacteriophage φVaK is clustered together with the VA1 prophage forming a separate monophyletic taxon (Figure 5).

The in vitro cell lysis experiment (Supplementary Figure S2) confirmed that the temperate bacteriophage H20 isolated as a free-living phage can be integrated in the host’s bacterial genome. The PCR with specific primers performed on the isolated bacterial strains was positive in amplifying the three selected gene parts of the H20-like phages. In 28% of the bacterial host strains (14 out of 50), the bacteriophage φΗ20 was successfully integrated after 10 h of co-culturing.

### 3.6. Distribution of H20-Like Phage on a Global Scale

**Figure 6:**
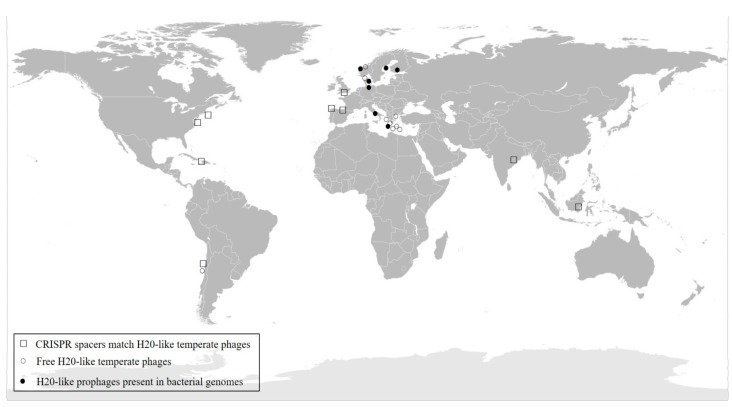
Distribution of H20-like phages on a global scale. The presence of the phage in the different locations was confirmed by: (a) isolating them as free phages from the environment (○); (b) bioinformatic detection of their presence as prophages in the bacterial host genomes (●); and (c) high similarity matches of the temperate phages with CRISPR spacers in the *Vibrio* genus (□).

Free-living H20-like bacteriophages have been isolated from Denmark, Norway, Greece and Chile. Combining these data with geographical distribution of the *Vibrio* CRISPR spacers matches and the functional prophages contained in the *V. anguillarum* genomes, the H20-like bacteriophages seem to show a global scale distribution (Figure 6).

## 4. Discussion

Several lytic Vibriophages have previously been sequenced and characterized in connection with their potential use as a strategy against vibriosis in aquaculture [61]. However, there are very few reports on temperate phages against non-cholera *Vibrio* strains [15,62] and their potential importance for lateral transfer of virulence and metabolic capacities among other *Vibrio* pathogens [11,20,63].

The H20-like phage genomes showed no similarity to available phage sequences in GenBank. However, all the phages displayed high genetic similarity with a sequence in in *V. anguillarum* strain NB10, which was then identified as a previously undetected prophage in genome of NB10 [49], and subsequently, in 17 other *V. anguillarum* strains. The most prominent differences between NB10 prophage and H20-like viruses were observed in the variable regions VR1, VR2 and VR3. 

Among the H20-like phages cluster, φVaK showed the largest genetic differences. Most of the genetic differences were synonymous, leaving the encoded genes unaltered. However, the variations of φVaK in the VR1 may explain the differences in its host range, since even small changes in structural proteins such as minor tail proteins and tail fiber proteins may affect host range [64]. The bacterial host strain VaKef that was used for the isolation of φVaK was also different from the closely related BA35 and T265 and A023 that were used for isolating the rest of the H20-like Siphoviruses. 

Phage tail length tape measure protein has a crucial role in phages’ genome injection into the bacterial host [65]. Interestingly, the structure of this protein in H20-like phages resembled the structure of the channel forming toxin colicin Ia [66], suggesting a dual role for the phage tail length tape measure gene. Since only one molecule of colicin Ia is needed to kill a bacterial cell (single hit kinetics) [67], these pore forming toxins are lethal for the bacteria [68,69]. A temperate phage encoded colicin Ia could therefore potentially confer a fitness advantage to its host against competing bacterial strains but could also be lethal for its own host. Lytic activity of phage tail length tape measure protein has previously been reported in *Staphylococcus aureus* specific Siphovirus vB_SauS-phiIPLA35, where a lysozyme-like domain of the tape measure protein had muramidase activity able to lyse *S. aureus* cells [70].

The detection of N6-adenine methyltransferase gene in the H20-like phages may contribute to explaining their wide distribution, as it potentially interferes with the Restriction-Modification (RM) bacterial phage defense system [71,72] thus improving the infection efficiency of the H20-like phages. RM systems generally consist of a restriction endonuclease which cleaves invading phage dsDNA and a methyltransferase, which catalyzes the methylation of specific bacterial dsDNA sequences, protecting the hosts own DNA from enzymatic cleavage. The presence of methyltransferase in the H20-like bacteriophages indicated that these phages can methylate their own DNA to avoid degradation by the host’s restriction enzymes. This finding is in accordance with other studies showing that the presence of virus-encoded N6-adenine methyltransferase can provide protection against restriction endonuclease activity [21,73]. However, the role of N6-adenine methyltransferase does not seem to operate solely as a counter defense mechanism to bacterial RM systems. Methyltransferases can also affect bacterial virulence [74] or function as transcriptional regulators by either activating or repressing bacterial genes [75,76]. N6-adenine methyltransferase was previously found in the temperate Vibriophage VHML where it was linked with the virulence of *V. harveyi* host strain upon integration [77]. Epigenetic control of gene expression through methylation is a powerful tool in prokaryotes emphasizing the potential influence of the H20-like phages in host functional properties. The presence of N6-adenine methyltransferase gene in H20-like phages genomes seems to be an important mutual benefit in the host-phage interaction which may promote coexistence. In general, methyltransferase-encoding genes are found in about 20% of the currently annotated bacteriophage genomes [78], supporting an important role of the gene for the phage–host interaction.

Examples of co-evolution of temperate bacteriophages and their bacterial hosts also includes phage-encoded prevention of infection by other phages by superinfection exclusion (Sie) [79]. The H20-like phages carry a repressor gene which genetically and structurally resembles the lambda temperate phage repressor. Such repressor proteins could potentially protect the lysogenized bacteria from similar superinfecting bacteriophages by blocking the expression of the lytic pathway genes [80,81] and this mechanism possibly confers repressor-mediated immunity to other H20-like phages in their *V. anguillarum* host. 

Prophages have previously been shown to potentially affect fitness and metabolic properties in fish pathogenic *Vibrios*. For example, the temperate Vibriophage VHML decreased the nutrient uptake in lysogenized *V. harveyi* cells, through a generalized suppression of metabolic activity as a potential energy-saving mechanism under nutrient-limited conditions [82]. Further, evidence of phage-encoded hemagglutinin, which is potentially involved in virulence of *V. pelagius* [63,83] and experimental verification of prophage-mediated virulence in *V. harveyi* [16] support that lysogenic conversion in *Vibrios* represents an important mechanism of adaptation to changing environmental conditions. The presence of functional genes in the H20 like-phages suggests that these phages may also represent a significant contribution to the phenotypic properties of *V. anguillarum* upon integration. 

The tRNA^Arg^ that was found in the genome of H20-like phages has also been reported in other Siphoviruses [84] and encodes the amino acid codon AGA which is considered a rare arginine tRNA [85]. Rare codons are generally responsible for encoding transcriptional and translational properties that are distinct from those encoded by the prevalent arginine codons and therefore affecting the expression of regulatory genes [86]. In accordance with this, all four arginine amino acids which are encoded by repressor gene in the H20-like bacteriophages are translated by the rare codon AGA. This suggests that the tRNA in the H20-bacteriophages participates in the regulation of the expression of the repressor, and thus in the decision of lytic or temperate life cycle, implying also that there might be other reasons for phages to carry tRNAs beyond rare codon usage. 

The isolation of phages and prophages belonging to the H20 group across large geographical scales and in marine environments with and without aquaculture activities suggests that these *V. anguillarum* phages are common and widespread in the marine environment. Further, the high levels of identity with spacers of the CRISPR systems detected in both *V. anguillarum* and in other *Vibrio* bacteria, indicated long term interactions between *Vibrios* and H20- like phages. This is in line with recent observations of susceptibility to specific phages in 36 isolates of the cosmopolitan Roseobacter-clade species, *Rugeria mobilis* obtained across the world’s oceans covering large ranges in temperature, oxygen concentration and habitat (free-living, particle attached, sediment) [25]. These results suggest the co-existence of specific phages and bacteria on a global scale in groups of ubiquitous marine bacteria such as *Roseobacter* and *Vibrio*. This seems inconsistent with the perception of phage–host interactions as a driver of phage and bacterial co-evolution and diversification, as this mechanism would be expected to promote local diversification in response to selection for phage resistance in bacterial populations. The ability of phages to move across biomes have shown to result in a high viral diversity on a local scale, but relatively low diversity when examined globally [28,87]. In addition, different types of phages seem to show different distribution patterns as phages belonging to Myoviridae and Podoviridae have demonstrated specific geographical distribution, whereas Siphoviruses displayed a global distribution [88].

The distribution patterns of specific phages and hosts and the implications of phage–host interactions on evolution and diversification thus seems to vary across spatial scales and between groups of bacteria and phages. While the co-evolutionary dynamics of phages and hosts have traditionally been characterized in terms of selection pressures on host defense and phage infectivity, the mutualistic nature of the interaction between temperate phages and their hosts may select for co-existence rather than arms race dynamics [20]. The efficient lysogenization of susceptible *V. anguillarum* by temperate phage H20 and thus transfer of phage-encoded genes between bacterial strains demonstrated in the current study emphasizes the potential of H20-like phages for integration in their host genome and thus the dispersal of the phage genes in *V. anguillarum*. Consequently, efficient dispersal of phages and bacteria across large spatial scales in the marine environment and a selective advantage of the phage–host interaction by lysogenic conversion of the host may select for phage–host co-existence in the global ocean and thus contribute to explaining the currently observed large scale distribution of H20-like temperate phages and prophages. 

## Figures and Tables

**Table 1:** Genomic information about the 19 sequenced bacteriophages of the current study. The bacteriophages were isolated against four different clinical *Vibrio anguillarum* strains (A023, T265, BA35, and VaKef). DK: Denmark, N: Norway, GR: Greece, CL: Chile, Aq: Aquaculture. M: Marine environment.

Bacteriophages	Origin	Environment	Host Strain	Genome Size (bp)	GC Content (%)	ORFs	GenBank Accession Number
φH2	DK	Aq	A023	46,149	43.1	76	KY658673
φH8	DK	Aq	T265	46,157	43.1	76	KY658674
φH20	DK	Aq	BA35	53,224	43	91	KY658675
φP2	N	Aq	BA35	46,149	43.1	76	KY658676
φP3	N	Aq	BA35	53,242	43	91	KY658677
pVa-1	GR	M	BA35	53,227	43.1	91	KX581095
pVa-2	GR	M	BA35	53,286	43	91	KX581094
pVa-3	GR	M	BA35	54,344	43.1	94	KY658678
pVa-4	GR	M	BA35	54,295	43.1	93	KY658679
pVa-5	GR	M	BA35	53,227	43	91	KX581096
pVa-6	GR	M	BA35	53,2274	43	91	KX581097
pVa-7	GR	M	BA35	54,268	43.1	93	KX581110
pVa-8	GR	M	BA35	53,227	43	91	KY658680
φCLA	CL	M	BA35	53,226	43	91	KX581091
φHer	GR	M	BA35	53,226	43	91	KX581090
φLen	GR	M	BA35	53,226	43	91	KX581092
φPel	GR	M	BA35	53,227	43	91	KX581093
φStrym	GR	M	BA35	53,226	43	91	KX581099
φVaK	GR	Aq	VaKef	53,216	43	92	KX581098

**Table 2:** Kinetic parameters (latency time, burst size and adsorption constant) of the bacteriophages φH20, pVa-3, φCLA and φP3. All values are means ± standard deviation of three independent experiments.

Bacteriophage	Latency Time (min)	Burst Size (Virions/Cell)	Adsorption Constant K_30_
φH20	60	112 ± 9	1.18 × 10^−7^ ± 1.28 × 10^−8^
pVa-3	60	100 ± 13	8.63 × 10^−8^ ± 2.46 × 10^−9^
φP3	70	101 ± 16	6.40 × 10^−8^ ± 4.39 × 10^−9^
φCla	50	145 ± 24	2.11 × 10^−7^ ± 2.68 × 10^−8^

**Table 3:** Bioinformatic analysis indicates the presence or absence of H20-like prophages in the genomes of 31 pathogenic *V. anguillarum* strains originated from several different aquaculture producing countries. n/a: not available.

Stain Code	Origin	Phylogenetic Group	H20-Like Prophage	Reference
DSM21597	Norway	I	-	[51]
4299	Norway	II	-	[52]
HI610	Norway	II	-	[53]
S2 2/9	Denmark	III	-	[54]
90-11-286	Denmark	III	-	[54,55]
PF430-3	Chile	IV	-	[36]
PF4	Chile	IV	-	[56]
PF7	Chile	IV	-	[56]
A023	Spain	V	X	[57]
90-11-287	Denmark	V	X	[55]
51/82/2	Germany	V	X	[57]
T265	UK	V	-	[57]
HWU53	Denmark	V	X	[57]
6018/1	Denmark	V	X	[57]
91-8-178	Norway	V	X	[57]
87-9-116	Finland	V	X	[54,55]
91-7-154	Denmark	V	X	[54,55]
601/90	Italy	V	X	[54]
178/90	Italy	V	X	[54]
9014/8	Denmark	V	X	[54]
261/91	Italy	V	X	[57]
LMG12010	n/a	V	X	[57]
87-9-117	Finland	V	X	[54,55]
BA35	USA	V	-	[57]
775	USA	V	-	[58,59]
NB10	North Baltic	V	X	[49,60]
VA1	Denmark	V	X	[36]
850610-1/6a	Denmark	n/a	X	[57]
VA2	Denmark	n/a	-	[36]
VA3	Denmark	n/a	-	[36]
VaKef	Greece	n/a	X	Clinical strain—Unpublished

## Supplementary Materials

The following are available online at www.mdpi.com/1999-4915/9/5/122/s1, Table S1: Three sets of specific primers designed for picking a hypothetical protein, a structural protein gene and a terminase protein gene, respectively. Table S2: Detailed annotations for the 19 temperate bacteriophages. Table S3: Spacers. Figure S1: One step growth curves and phage adsorption curves, for bacteriophages φH20, pVa-3, φCLA and φP3. Figure S2: In vitro cell lysis experiment of bacteriophage φH20 against its bacterial host V. anguillarum strain BA35. Arrow indicates the sampling point.
