# Supplementary material for: Stumbling across the Same Phage: Comparative Genomics of Widespread Temperate Phages Infecting the Fish Pathogen Vibrio anguillarum
Source: Viruses. 2017 May 20;9(5):122. doi: 10.3390/v9050122 (PMC5454434; doi:10.3390/v9050122)
Supplement: Supplementary file 1 [file viruses-09-00122-s001.zip › Supplementary Figure 2.docx]

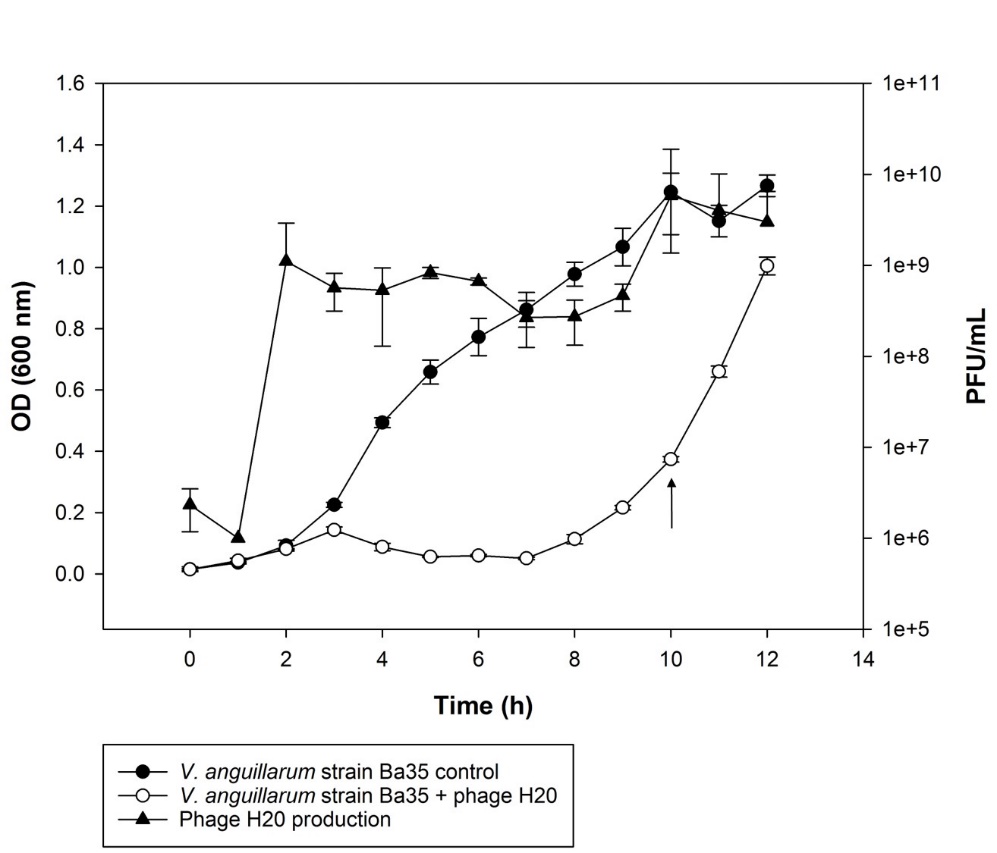


**Supplementary Figure 2** In vitro cell lysis experiment of bacteriophage φH20 against its bacterial host V. anguillarum strain BA35. Arrow indicates the sampling point.
