# Supplementary material for: Stumbling across the Same Phage: Comparative Genomics of Widespread Temperate Phages Infecting the Fish Pathogen Vibrio anguillarum
Source: Viruses. 2017 May 20;9(5):122. doi: 10.3390/v9050122 (PMC5454434; doi:10.3390/v9050122)
Supplement: Supplementary file 1 [file viruses-09-00122-s001.zip › Supplementary Table 1.docx]

**Supplementary Table 1** Three sets of specific primers designed for picking a hypothetical protein, a structural protein gene and a terminase protein gene, respectively.

| **Forward primer** | **Reverse primer** | **Target gene** | **Product size (bp)** |
| --- | --- | --- | --- |
| CAAAGGTGAACATGGGCGTG | CGGAAGGAATGGGCAGTTCT | Hypothetical protein (HP) | 222 |
| TGGGACTTTCGCTCAGGAAC | TGGGTAGCGCATTTTACGGT | Structural protein (SP) | 447 |
| TACCACGAACACGAAAGCGA | GATAGTCAACCTCGGCTCCG | Terminase protein (TP) | 673 |
