# Supplementary material for: Stumbling across the Same Phage: Comparative Genomics of Widespread Temperate Phages Infecting the Fish Pathogen Vibrio anguillarum
Source: Viruses. 2017 May 20;9(5):122. doi: 10.3390/v9050122 (PMC5454434; doi:10.3390/v9050122)
Supplement: Supplementary file 1 [file viruses-09-00122-s001.zip › Supplementary Table 3.docx]

**Supplementary Table 3** The spacers of **A.** CRISPR array 1 of V. anguillarum PF7 (spacer59, spacer5: no match with φΗ2, φΗ8, φP2 – spacer67, spacer59: 91.2% and no match, respectively, with φVaK) , **B.** CRISPR array 2 of V. anguillarum PF7 (spacer67: no match with φΗ2, φΗ8, φP2 – spacer66, spacer67: 97.1% and no match, respectively, with φVaK) and **C.** CRISPR arrays of the rest 63 Vibrio species, that were mapped to the H20-like phages. Cut-off value was set at 80%.

**A.**

| **Spacer description** | **% nt identity** | **Spacer sequence** |
| --- | --- | --- |
| spacer68 | 100 | AAUUGAAGAUCACUCAAGCUCAACACAAAGAGG |
| spacer72 | 91.2 | CUGAACGCCGCAAUAAUCGGAGACGCAACCAUCA |
| spacer67* | 97.1 | UUGACGAAAGAAACCGAGCGCCAGAAUUAGAGCU |
| spacer64 | 100 | CAUCUUGUCGAUGCGGUCAUAUUCAGCAACAAC |
| spacer75 | 85.3 | UUUACCGGGGCCAGUUAAUACCUCGGCAGGAAUG |
| spacer39 | 100 | CGCGAAUGUAGCUUUCUGCGUCCAUCAUGCGAG |
| spacer60 | 97 | CGCGAAUAUAGCUUUCUGCGUCCAUCAUGCGAG |
| spacer61 | 100 | CGGGCACAUAACAUUGGUUACUAAGCCAAAGUU |
| spacer37 | 97.1 | UCUUGCCGCUGUUAUGAUCCCGUUUGACAAGCAA |
| spacer59* | 100 | UCCGAUUAAGUCGAUAGCAGCCAAUACCUUUGCU |
| spacer69 | 97 | CTCAAGTCATCGAGGCTGGTGGTGTGAAACTGA |
| spacer70 | 100 | AUGCUCGGCUAUGAACUCUUGCCAAGCUCCGAU |
| spacer74 | 100 | AUGAGCUGCGCAGUUCGCUUGACUGUUUCAAGC |
| spacer5 | 81.1 | CTGCCAGCGATCAACTAATGCTGCCGTAAATTC |

**B.**

| **Spacer description** | | | **% nt identity** | | **Spacer sequence** | | | |  |
| --- | --- | --- | --- | --- | --- | --- | --- | --- | --- |
| spacer75 | | | 97 | | UGGGGAUAAUCUAGACUUUAUGCACAUCGAGGG | | | |  |
| spacer72 | | | 97 | | AGAAAUGAAGAUUUACCCAAAGCAAAAAACUUA | | | |  |
| spacer73 | | | 100 | | AAAAGCCAAGCAGAUAUUUUUACUGGCUGGCAG | | | |  |
| spacer66* | | | 100 | | CAUGUUCACAGCAGCAAGAGAGACUAGCUCUAUU | | | |  |
| spacer77 | | | 97 | | AUGGUCUGAUCGUCUCGGCUUUCGCCUCGGUAG | | | |  |
| spacer65 | | | 97 | | UACUGAUUCAAUGUUAAAAACCUGCCCUUCCCA | | | |  |
| spacer74 | | | 85.7 | | AAAUGGUUCAGAGCGUGGCGUUUUUAGCAAGUUCG | | | |  |
| spacer67* | | | 85.3 | | CCUAUGGAACGCCAACAGACUCAAGAGAUCGACC | | | |  |
| spacer76 | | | 97.1 | | UGGGAAACAGUAAAACAGAUGUCGACACUGACAG | | | |  |
| spacer69 | | | 90.9 | | AGCCAUUGAUAAGUGUCCGCAUACAUUUUCAAG | | | |  |
|  | | |  | |  | | | |  |
| **C.** | ***Vibrio species*** | **Strain** | | **Origin** | | **Spacer description** | **% nt identity** | **Spacer sequence** | |
|  | *Vibrio cholerae* | 1157-74 | | India | | spacer41 | 93.8 | AAAGCCCTTCGGTAGCGAA-AGGAAAGACTCT | |
|  | *Vibrio cholerae* | RC 27 | | Indonesia | | spacer40 | 81.8 | GACCTGATTTACGTGCGAGAGACGTTTCGCCTG | |
| (2x) | *Vibrio cholerae* | 984-81 | | India | | spacer5 | 81.8 | TCGAGAGTCGTTAGGTTCTTGTCGCAGTATTCG | |
| (2x) | *Vibrio cholerae* | 984-81 | | India | | spacer6 | 90.9 | GCCAAGCGCTCTTCGGCACGCAGCGTAACCATG | |
|  | *Vibrio navarrensis* | 2232 | | Spain | | spacer7 | 87.5 | CCGCCACCACCGCCACCGCCAGCGGTTTTTAA | |
|  | *Vibrio navarrensis* | ATCC 51183 | | Spain | | spacer21 | 100 | TGTTAGCCACTCCAGACCGCTTTCAAAATACG | |
|  | *Vibrio navarrensis* | ATCC 51183 | | Spain | | spacer6 | 90.9 | TCTTCATCGCTTAGTCCTTTCTGATTTGTGACT | |
|  | *Vibrio metoecus* | YB5B04 | | USA | | spacer36 | 84.4 | AGCAGCCGCTTGTTGTGAGGCAATTCTCGCCA | |
